# Upregulation of cystathionine beta-synthetase expression by nuclear factor-kappa B activation contributes to visceral hypersensitivity in adult rats with neonatal maternal deprivation

**DOI:** 10.1186/1744-8069-8-89

**Published:** 2012-12-18

**Authors:** Lin Li, Ruihua Xie, Shufen Hu, Yongmeng Wang, Tianzhu Yu, Ying Xiao, Xinghong Jiang, Jianguo Gu, Chuang-Ying Hu, Guang-Yin Xu

**Affiliations:** 1 Department of Gastroenterology, the Second Affiliated Hospital, Soochow University, Suzhou, 215123, P. R. China; 2 Institute of Neuroscience, Key Laboratory of Pain Basic Research and Clinic Therapy, Department of Neurobiology, Soochow University, Suzhou, 215123, P R China; 3 Department of Anesthesiology and the Graduate Program in Neuroscience, The University of Cincinnati College of Medicine, PO Box 670531, 231 Albert Sabin Way, Cincinnati, OH, 45267, USA

**Keywords:** Dorsal root ganglion, Neonatal maternal deprivation, Chronic visceral pain, Hydrogen sulfide, Nuclear factor kappa B

## Abstract

**Background:**

Irritable bowel syndrome (IBS) is characterized by chronic visceral hyperalgesia (CVH) that manifested with persistent or recurrent abdominal pain and altered bowel movement. However, the pathogenesis of the CVH remains unknown. The aim of this study was to investigate roles of endogenous hydrogen sulfide (H_2_S) producing enzyme cystathionine beta-synthetase (CBS) and p65 nuclear factor-kappa B subunits in CVH.

**Results:**

CVH was induced by neonatal maternal deprivation (NMD) in male rats on postnatal days 2–15 and behavioral experiments were conducted at the age of 7–15 weeks. NMD significantly increased expression of CBS in colon-innervating DRGs from the 7^th^ to 12^th^ week. This change in CBS express is well correlated with the time course of enhanced visceromoter responses to colorectal distention (CRD), an indicator of visceral pain. Administration of AOAA, an inhibitor of CBS, produced a dose-dependent antinociceptive effect on NMD rats while it had no effect on age-matched healthy control rats. AOAA also reversed the enhanced neuronal excitability seen in colon-innervating DRGs. Application of NaHS, a donor of H_2_S, increased excitability of colon-innervating DRG neurons acutely dissociated from healthy control rats. Intrathecal injection of NaHS produced an acute visceral hyperalgesia. In addition, the content of p65 in nucleus was remarkably higher in NMD rats than that in age-matched controls. Intrathecal administration of PDTC, an inhibitor of p65, markedly reduced expression of CBS and attenuated nociceptive responses to CRD.

**Conclusion:**

The present results suggested that upregulation of CBS expression, which is mediated by activation of p65, contributes to NMD-induced CVH. This pathway might be a potential target for relieving CVH in patients with IBS.

## Background

Irritable bowel syndrome (IBS) is a common gastrointestinal disorder characterized by abdominal pain in association with altered bowel movements without a demonstrable pathology. The visceral hypersensitivity is an important neurophysiological mechanism in patients with IBS [1]. Recent studies in rodents found that early life stress by neonatal maternal deprivation (NMD) could induce intestinal mucosal dysfunction and visceral hypersensitivity at the adult stage in rats, mimicking main pathophysiological characteristics of IBS in human [2,3]. Indeed, early traumatic experiences such as childhood neglect, abuse, loss of a parent, and life-threatening situations during childhood have been shown to increase the risk of IBS development in human [4]. The NMD-induced visceral hypersensitivity is distinct from those of inflammatory pain and neuropathic pain in that it produces visceral hyperalgesia without inflammatory responses in the gut mucosa, a characteristic of IBS. Unfortunately, visceral pain of IBS cannot be effectively treated at the present time partially because its pathophysiology is not well understood.

Progress has been made during last several decades in understanding the development of visceral hypersensitivity. Hydrogen sulfide was discovered to be one of principal molecules to modulate nociceptive processes in primary afferents including those that innervate visceral organs. It has been recently shown that cystathionine *β*-synthetase (CBS) signaling pathway plays an important role in the induction of both acute and chronic pain. H_2_S participated in formalin-induced peripheral inflammatory pain in hindpaws of rats [5]. It also enhanced carrageennan-induced hindpaw oedema in the rats, and this effect may be mediated by increasing the activity of myeloperoxidase. Both intrathecal and intraplantar injection of NaHS activated T-type Cav3.2 and then lead to peripheral mechanical hyperalgesia [6,7]. H_2_S contributes to the generation of visceral hyperalgesia not only in the peripheral somatosensory system but also in the visceral organs [8]. Since CBS is the predominant source of H_2_S synthesis in the colon of rodents [9], we investigated function and regulation of CBS-H_2_S signaling pathway in the NMD-induced visceral hyperalgesia.

Emerging evidence has also indicated that the activation of nuclear factor kappa B (NF-κB) following peripheral inflammation or nerve injuries is related to the generation of hyperalgesia or allodynia [10,11], but its roles in CHV has not been fully studied. Chronic nerve injury could result in an increase in the expression of phospho-NF-κB (p-NF-κB) in astrocytes and p-NF-κB was shown to participate in tactile allodynia [12-14]. During peripheral and spinal nerve injuries, activity of NF-κB was increased in injury-related DRGs and spinal segments [15-17]. Intraneural injection of interleukin-1beta and tumor necrosis factor-alpha into rat sciatic nerve at physiological doses evoked signs of neuropathic pain [18]. Intrathecal pretreatment with NF-κB inhibitors attenuated the allodynia produced by sciatic neuropathy [11,15,19-22]. These lines of evidence indicate that NF-κB, a classic transcription factor, is a key regulator of genes involved in the response to inflammation and stress [23-25]. Previous studies have mainly focused on the regulation of NF-κB in gene expression of pro-inflammatory cytokines such as TNF-α, IL-1β, IL-6 and prostaglandins [11,26,27]. Attention has not been well paid to the action of NF-κB on CBS-H_2_S signaling, a pathway that is particularly important for the generation of visceral hypersensitivity and chronic visceral pain.

As an extension of our previous work in peripheral sensitization of NMD-induced visceral hyperalgesia, in this study, we focused on changes in expression and function of endogenous H_2_S-producing enzyme CBS and NF-κB in colon-innervating DRGs in adult rats with NMD. Our findings indicate that upregulation of CBS expression mediated by translocation of p65 from cytoplasm to nucleus contributes to NMD-induced CVH. This provides a mechanistic insight into CVH from a neurobiological perspective.

## Methods

### Induction of chronic visceral hypersensitivity

Experiments were performed on male Sprague–Dawley rats. Care and handling of all animals were approved by the Institutional Animal Care and Use Committee of Soochow University and were in accordance with the guidelines of the International Association for the Study of Pain. Chronic visceral hypersensitivity (CVH) was induced by neonatal maternal deprivation (NMD), a rat model described previously [2,28]. Briefly, pups were separated from their dams for 3 hours once a day on postnatal day 2–15 with the temperature maintained at ~32°C. After the separation period, pups were returned to their maternity cages housed as a group. Pups in no handing group were raised with their dams as age- and sex-matched controls (CON). On PND 21~22, pups were weaned and housed in individual cages in the same way for both CON and NMD groups. All experiments were performed at the age of 7–15 weeks. In the present study, a total of 200 male rats were used, of which 83 rats was in CON group (n = 40 for behavioral studies, n = 14 for excitability recordings, n = 29 for Western blotting), and 117 rats (n = 72 for behavioral studies, n = 11 for excitability recordings, n = 34 for Western blotting) in NMD group.

### Behavioral studies to measure nocifensive responses to graded CRD

Visceral hypersensitivity was measured at postnatal 7–15 weeks by observing the response of rats to colorectal distention (CRD), as described previously [29,30]. In brief, a flexible balloon (6 cm) constructed from a surgical glove finger attached to a tygon tubing was inserted 8 cm into the descending colon and rectum via the anus and held in place by taping the tubing to the tail. Rats were placed in small Lucite cubicles and allowed to adapt for 30 minutes. CRD was performed by rapidly inflating the balloon to a constant pressure measured using a sphygmomanometer connected to a pressure transducer. The balloon was inflated to various pressures: 20, 40, 60 and 80 mmHg, for a 20 second stimulation period followed by a 2 minute rest. Behavioral responses to CRD were measured by visual observation of the abdominal withdrawal reflex (AWR) by a blinded observer and the assignments of an AWR score were as follows: 0=no response to given pressure; 1=slight head movement without abdominal muscle contraction; 2=Contraction of abdominal muscles; 3=lifting of abdominal wall; 4=Body arching and lifting of pelvic structures. The experimenter, who assigned the AWR scores and performed the EMG analysis, was masked to the control or NMD group assignment and the drug treatment group as well.

To minimize the possible insult from the repetitive distention stimuli of the colon, distention threshold was measured in this study. Distension threshold (DT) was the minimal distention pressure to evoke abdominal visceromotor response. It was recorded in mmHg by giving a steady increase in distention pressure by sphygmomanometer.

### Cell fractionation

Owing to small content of nuclear protein in one DRG, proteins of 24 DRGs from 4 rats from each group were pooled together as one sample. Thus total twelve rats from CON and NMD group were used in this experiment. Nuclear protein was extracted by NE-PER nuclear and cytoplasmic extraction reagents (Thermo) according to manufactures instruction. Briefly, immediately before use, 1% (v/v) protease inhibitors (Roche) were added to the reagents. Twenty milligram DRGs were homogenized using glass grinder with the indicated 200 μl cytoplasmic extraction reagent and vortexed vigorously for 15s and then incubated on the ice for 10min. Next 11μl of the second regent was added to the suspension for 1 min. The suspension was centrifuged at 16000 rpm for 5 min at 4°C. The supernatant (cytoplasmic fraction) were collected and stored at −80°C. Sedimented nuclei were gently resuspended in 100 μl of ice-cold nuclear extraction regent and 1% (v/v) protease inhibitors, incubated on ice, vortexed for 15s every 10 min, for a total of 40 min. The suspension was centrifuged at 16000 rpm for 10 min at 4°C. The supernatant (nuclear fraction) was transferred to a pre-chilled tube and stored at −80°C until use. Protein concentration was determined by the Bradford method (Beyotime).

### Western blot

DRGs (T_13_-L_2_) from control rats or NMD rats were dissected out and lyzed in 100 μl of radioimmunoprecipitation assay buffer containing 1% NP-40, 0.5%Na deoxycholate, 0.1% SDS, PMSF (10 μl/ml), and aprotinin (30 μl/ml; Sigma). The cell lysates were then microfuged at 15,000 rpm for 30 min at 4°C. The concentration of protein in homogenate was determined using a BCA reagent (Beyotime, CHN). Twenty micrograms of proteins were loaded onto a 10% Tris–HCl SDS-PAGE gel (Bio-Rad, Hercules, CA). After electrophoresis, the proteins were electrotransferred onto polyvinylidenedifluoride membranes (Millipore) at 200 mA for 2 hr at 4°C. The membranes were incubated in 25 ml of blocking buffer (1XTBS with 5% w/v fat-free dry milk) for 2 hr at room temperature. The membrane was then incubated with the primary antibodies at 4°C overnight. Primary antibodies used were mouse anti-CBS (1:1000; Abnova, Taiwan, CHN) and mouse anti-NF-κB (1:200; Santa Cruz, California, USA). After incubation, membranes were washed with TBST (1XTBS and 0.5% Tween-20) three times for 15 min each and incubated with anti-mouse HRP-conjugated secondary antibody (1:4000; Chemicon) for 2 hr at room temperature. The membrane was then washed with TBST three times for 15 min each. The immunoreactive proteins were detected by enhanced chemiluminescence (ECL kit; Amersham Biosciences, Arlington Heights, IL). Bands recognized by primary antibody were visualized by exposure of the membrane onto an x-ray film. Then membrane was striped and re-probed for β-actin (anti-actin 1:1000, Multisciences, CHN) or H_3_ (anti-H_3_, 1:1000, Sigma, USA). For quantification of the objective protein levels, the photographs were digitized and analyzed using a scanner (Bio-Rad imaging system Bio-Rad GelDoc XRS+). All samples were normalized to β-actin or H_3_ as control.

### Cell labeling

Colon specific DRG neurons were labeled by injection of 1, 1'-dioleyl-3, 3, 3', 3-tetramethylindocarbocyanine methanesulfonate (DiI, Invitrogen) into the colon wall [30]. Briefly, when the rats were 6 weeks old, animals were anaesthetized with ketamine (80 mg/kg, i. p.) plus xylazine (5~10 mg/kg, i. p.). The abdomen was opened by midline laparotomy and the colon was exposed. DiI, 25 mg in 0.5 ml methanol, was injected in ~1 μl volume at 10 sites on the exposed colon extending from the level of the bladder to about 6 cm in an oral direction. To prevent leakage and possible contamination of adjacent organs with the dye, the needle was left in place for 1 min and each injection site was washed with normal saline following each injection. The colon was gently swabbed prior to closing of the abdomen. Animals were returned to their cages and given free access to drinking water and standard food pellets.

### Whole-cell patch clamp recordings

Ten days after DiI injection, NMD or control rats were killed by cervical dislocation, followed by decapitation [31]. Bilateral DRGs (T_13_-L_2_) were dissected out and transferred to an ice-cold, oxygenated fresh dissecting solution, containing (in mM): 130 NaCl, 5 KCl, 2 KH_2_PO_4_, 1.5 CaCl_2_, 6 MgSO_4_, 10 glucose and 10 HEPES, pH 7.2 (osmolarity: 305 mOsm). After removal of the connective tissue, the ganglia were transferred to a 5 ml dissecting solution containing collagenase D (1.8-2.0 mg/ml; Roche, Indianapolis, Indiana, USA) and trypsin (1.2-1.5 mg/ml; Sigma, St Louis, Missouri, USA) and incubated for 1.5 h at 34.5°C. DRGs were taken from the enzyme solution, washed and transferred to 0.5 ml of the dissecting solution containing DNase (0.5 mg/ml; Sigma, St Louis, Missouri, USA). A single cell suspension was subsequently obtained by repeated trituration through flame-polished glass pipettes. Cells were plated onto acid-cleaned glass coverslips. Coverslips containing adherent DRG cells were put in a small recording chamber (1 ml volume) and attached to the stage of an inverted microscope (Olympus IX71) fitted for both fluorescence and bright-field microscopy. DiI-labeled neurons were identified by their fluorescence under the fluorescent microscope. For the patch-clamp recording experiments, cells were continuously superfused (1.5 ml/min) at room temperature with normal external solution containing (in mM): 130 NaCl, 5 KCl, 2 KH_2_PO_4_, 2.5 CaCl_2_, 1 MgCl_2_, 10 HEPES, 10 glucose, with pH adjusted to 7.2 with NaOH, osmolarity: 295–300 mOsm). Recording pipettes were pulled from borosilicate glass tubing using a horizontal puller (P-97, Sutter Instruments). Unless indicated, patch-clamp pipettes had a resistance of 4–7 MΩ when filled with the pipette solution containing (in mM): 140 potassium gluconate, 10 NaCl, 10 HEPES, 10 glucose, 5 EGTA and 1 CaCl_2_, pH = 7.25 adjusted with KOH; osmolarity: 292 mOsm. The voltage was clamped at −60 mV by a HEKA EPC10 patch clamp amplifier (HEKA; Germany). Capacitive transients were corrected using capacitive cancellation circuitry on the amplifier that yielded the whole cell capacitance and access resistance. Up to 90% of the series resistance was compensated electronically. Considering the peak outward current amplitudes of smaller than 10 nA, the estimated voltage errors from the uncompensated series resistance would be smaller than 10 mV. The leak currents at −60 mV were always smaller than 20 pA and were not corrected. The currents were filtered at 2–5 kHz and sampled at 50 or 100 μs/point. Whole cell current and voltage were recorded with a HEKA EPC10 patch-clamp amplifier; and data were acquired and stored on a computer for later analysis using FitMaster (HEKA; Germany). All experiments were performed at room temperature (~22°C).

### Drug application

O-(Carboxymethyl) hydroxylamine hemihydrochloride (AOAA, an inhibitor of CBS), NaHS and pyrrolidine dithiocarbamate (PDTC, an inhibitor of p65) were purchased from Sigma-Aldrich and were freshly prepared in 0.9% normal saline. To keep consistency, AOAA were intraperitoneally injected 30 min before behavioral measurement or injected once daily for consecutive 7 days for molecular expression experiments as described previously [8,30]. PDTC was intrathecally injected once for behavioral test or once daily for continuous 7 days for detection of target protein expression. NaHS at different doses was injected intrathecally.

### Data analysis

All data are expressed as mean ± SEM. Statistical analysis were conducted using commercial software OriginPro 8 (OriginLab, US) and Matlab (Mathworks, US). Normality was checked for all data before analyses. Significance was determined using paired sample *t*-Test, paired sample sign test, two sample t test, Mann–Whitney test, Tukey post hoc test following Kruskal-Wallis ANOVA or two-way repeated-measures ANOVA, Mann–Whitney test following Friedman ANOVA, as appropriate. The level of significance was set at p < 0.05.

## Results

### NMD increases CBS expression in an association with enhanced visceral hypersensitivity

**Figure 1:**
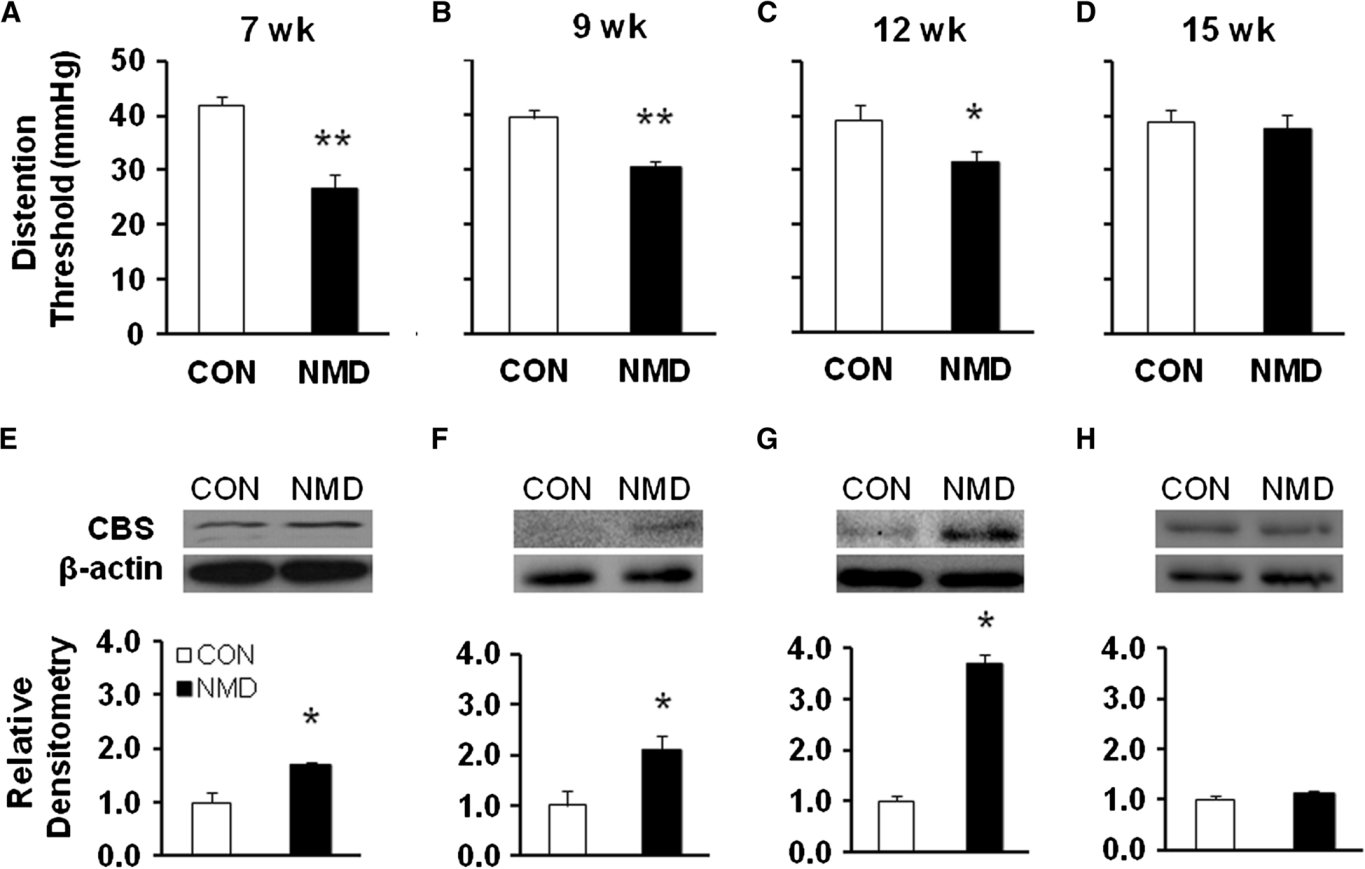
**NMD enhanced CBS expression in rats with visceral hypersensitivity.** (**A**-**D**). Time course of changes in distention threshold after NMD. NMD significantly reduced distention threshold when compared with age-matched controls at the 7 weeks (**A**, n=8 rats for each group, **P<0.01), 9 weeks (**B**, n=8 for each group, **P<0.01), 12 weeks (**C**, n=8 rats for each group, *P<0.05). NMD did not significantly alter distention threshold when compared with age-matched controls at the 15 weeks (**D**, n=8 rats for each group). (**E**-**H**). Time course of changes in CBS expression after NMD. NMD significantly upregulated expression of CBS and increased AWR scores when compared with age-matched controls at 7 weeks (**E**, n=3 rats for each group; *P< 0.05), 9 weeks (**F**, n=3 rats for each group, *P<0.05), and 12 weeks (**G**, n=3 rats for each group; *P<0.05). NMD did not significantly alter CBS expression when compared with age-matched controls at 15 weeks (**H**, n=3 rats for each group). Actin was used as an internal control for each group.

Chronic visceral hyperalgesia (CVH) was determined by measuring the distention threshold (DT) in response to CRD at 7–15 weeks of age. The DT was significantly lower in NMD-treated rats at 7 weeks (Figure 1A, **p < 0.01, n=8 for each group, two sample t test), 9 weeks (Figure 1B, **p < 0.01, n=8 for each group, two sample t test), 12 weeks (Figure 1C, *p < 0.05, n=8 for each group, two sample t test) than those in age matched control (CON) rats, respectively. The reduced DTs lasted for ~6 weeks and returned to the normal level at 15 (Figure 1D, n=8 for each group) weeks of age, which is consistent with our previous report that NMD significantly increased AWR scores to CRD in adult rats compared with age-matched controls [32].

To determine the effect of NMD on CBS expression, bilateral thoracolumbar (T_13_, L_1_, L_2_) DRGs were dissected out at 7, 9, 12 and 15 weeks. NMD remarkably increased CBS expression in T_13_-L_2_ DRGs at 7, 9 and 12 weeks when compared with age-matched controls. CBS expression was returned to the normal level 15 weeks after birth. The ratios (NMD/CON) of relative densitometry were 1.69 (Figure 1E, n=3 for each group, *p < 0.05, two sample t test), 2.11 (Figure 1F, n=3 for each group, *p < 0.05, two sample t test), 3.69 (Figure 1G, n=3 for each group, *p < 0.05, two sample t test) and 1.13 (Figure 1H, n=3 for each group, p >0.05) for 7, 9, 12 and 15 weeks, respectively. Thus, NMD treatment upregulated CBS expression in colon related DRGs, suggesting a correlation between the enhanced visceral sensitivity and CBS expression in terms of time course in NMD rats.

### CBS inhibitor AOAA attenuates AWR scores in NMD rats

**Figure 2:**
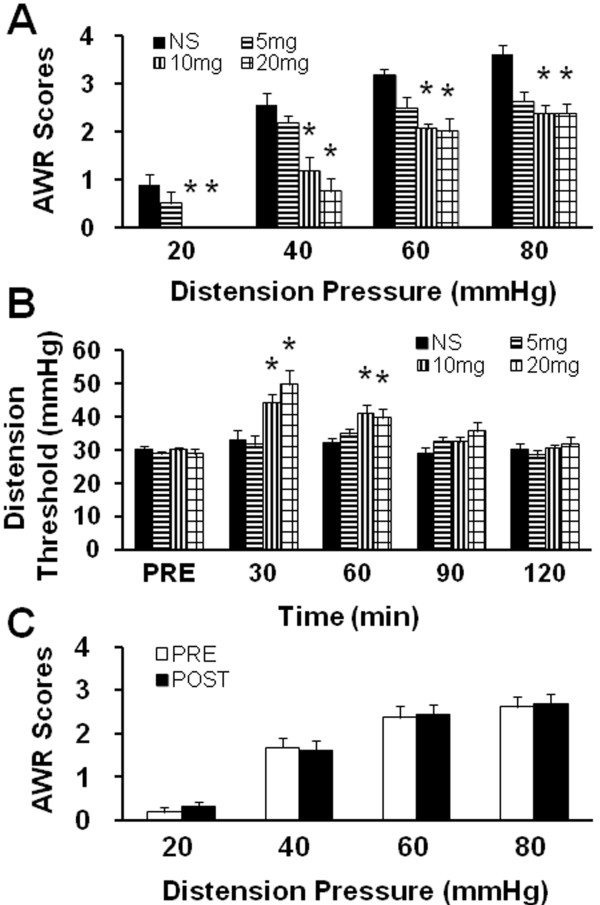
**CBS inhibitor AOAA attenuated AWR scores in NMD rats.** (**A**) CBS inhibitor abolished visceral hyperalgesia induced by NMD in a dose dependent manner. The maximal effect was at 10 mg/kg body weight (i.p.) in this study. n=8 rats for each dose group; *P<0.05. (**B**) CBS inhibitor mitigated visceral hyperalgesia induced by NMD time-dependently. AOAA-induced antinociceptive effect lasted for about 60 minutes in both 10 and 20 mg/kg body weight. n=8 rats for each group; *P<0.05. (**C**) AOAA (10 mg/kg body weight) had no effect on AWR scores of age-matched healthy control rats (n=8).

To determine whether CBS activity is involved in NMD-induced visceral hypersensitivity, CBS inhibitor, AOAA, was administrated intraperitoneally (i.p.). Injection of AOAA significantly reduced AWR scores in NMD rats, in a dose-dependent fashion (Figure 2A, n = 8 for each group; *p < 0.05 vs. NS, Tukey post hoc test following Kruskal-Wallis ANOVA). The optimized dose for AOAA to produce the maximal effect was 10 mg/kg body weight in this study. We then determined the time course of AOAA effects. The effect of AOAA at doses of 10 and 20 mg/kg body weight lasted for ~60 min (Figure 2B, n = 8 for each group; *p < 0.05 vs. NS, Tukey post hoc test following two-way repeated-measures ANOVA). Maximal inhibition was at 30 min. To further confirm the AOAA effect on NMD rats, AOAA at 10 mg/kg was i.p. injected into age-matched healthy control rats. Application of AOAA had no significant effects on AWR scores in healthy control rats (Figure 2C, n = 8 for healthy group). These data suggest that this agent did not act as a non-specific analgesic and that CBS do not normally participate in the responses to CRD in rats under normal conditions.

### CBS inhibitor AOAA reverses hyperexcitability of colon-innervating neurons

**Figure 3:**
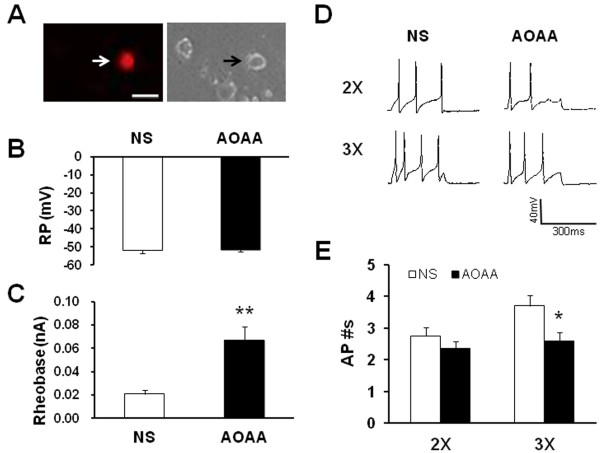
**CBS inhibitor AOAA reduced neuronal excitability.** (**A**). An example of a Dil-labeled DRG neuron (Left, arrow). A phase image of the same DRG neuron is shown on the right (arrow). Bar=50 μm. Patch clamp recordings were made from Dil-labeled neurons isolated from T_13_-L_2_ DRGs. AOAA was intraperitoneally injected at the dose of AOAA 10 mg/kg body weight once a day for consecutive 7 days. (**B**) Administration of AOAA did not produce any effect on resting membrane potentials (RP). n=20 cells for each group. (**C**) Administration of AOAA, however, significantly increased rheobase when compared with NS-treated NMD rats. n=20 cells for each group; **P<0.01. (**D**) Representative traces of APs were induced by 300 ms depolarizing current pulses injected through the patch pipette at 2 times and 3 times rheobase in DiI labeled neurons from NS- (left) and AOAA- (right) treated NMD rats under current-clamp conditions. (**E**) Bar graph showed the average number of APs evoked by 3 times rheobase current stimulation was significantly reduced while the average number of APs evoked by 2 times rheobase current stimulation was not significantly reduced when compared with NS group. n=20 cells for each group; *P<0.05.

Since AOAA attenuated the AWR scores in NMD rats, we next investigated whether AOAA suppressed excitability of DiI-labeled DRG neurons. To determine the effect of AOAA on neuronal excitability, NMD rats were divided into two groups: AOAA group treated with AOAA (10 mg/kg, i.p.) and NS group treated with the same volume of normal saline (NS, i.p.). Colon specific DRG neurons (Figure 3A) were harvested 4.5 hours after termination of the last injection. AOAA treatment did not significantly change the resting membrane potentials (Figure 3B). The resting membrane potentials were −51.90±1.73 (n=20 cells) and −51.45±1.33 (n=20 cells) for NS and AOAA, respectively. However, AOAA treatment dramatically enhanced rheobase when compared with the NS-treated group (Figure 3C, NS: 0.02 ± 0.003 nA; AOAA: 0.07 ±0.01 nA, n =20 cells for each group, **p < 0.01. Mann–Whitney test). In addition, AOAA injection markedly decreased the number of action potentials (APs) evoked by 3X rheobase current stimulation (Figure 3D and E), but not by 2X rheobase current stimulation. The average numbers of APs evoked by 2X rheobase stimulation were 2.75 ± 0.26 and 2.35 ± 0.22 for NS and AOAA (n=20 cells for each group), respectively (Figure 3E, left, p>0.05). The average numbers of APs evoked by 3X rheobase stimulation were 3.70 ± 0.33 and 2.60 ± 0.28 for NS and AOAA (n=20 cells for each group), respectively (Figure 3E, * p < 0.05, Mann–Whitney Test).

### Addition of NaHS enhances neuronal excitability

**Figure 4:**
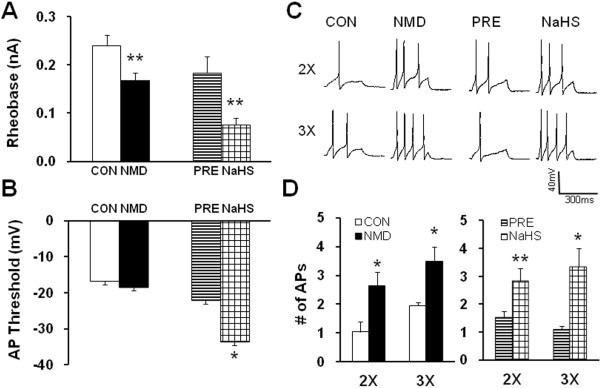
**NaHS enhanced neuronal excitability.** (**A**) NMD significantly decreased the rheobase comparing with CON. n=28 cells for CON, n=41 cells for NMD. **P<0.01. Similarly, NaHS application significantly decreased the rheobase comparing with PRE. n=13 cells, **P<0.01. (**B**) NMD failed to change AP threshold compared with control. n=41 and 27 cells for NMD and CON, respectively. However, NaHS application markedly hyperpolarized AP thresholds. n=13,*P<0.05. (**C**) Representative traces of APs were induced by 300 ms depolarizing current pulses at 2 times and 3 times rheobase in DiI labeled neurons from control and NMD rats (far two left traces) and DiI labeled neurons from healthy control rats before and after NaHS application (far two right traces) under current-clamp conditions. (**D**) Bar graph showing that NMD treatment significantly increased average number of APs elicited by a 2 or 3 times rheobase current injection of DiI neurons compared with controls (left). n=25 and 21 cells for CON in 2 times and 3 times rheobase stimuli, respectively, n=40 and 36 cells for NMD in 2 times and 3 times rheobase stimuli, respectively. *P<0.05. Similarly, application of NaHS remarkably increased the number of APs evoked by 2 and 3 times current stimulation (right). n=13 cells for each group in 2 times rheobase stimuli, n=9 cells for each group in 3 times rheobase stimuli. *P<0.05, **P<0.01.

Since the production of CBS enzyme is H_2_S, we then determined the role for H_2_S on neuronal excitability. NaHS, a donor for H_2_S, was applied in the study. Application of NaHS is to mimic CBS production of H_2_S. The reason to apply NaHS is because its use enables us to define H_2_S concentrations in the solution more accurately and reproducibly than directly bubbling H_2_S gas. In the neutral pH solution, one-third of NaHS exists as H_2_S and the remaining two-thirds are present as HS^−^. To better compare the effect of H_2_S and NMD-induced effect on neuronal excitability, we firstly examined the rheobase, AP threshold and numbers of AP evoked by 2X and 3X rheobase current stimulation in NMD rats. In an agreement with our previous report [33], NMD treatment markedly reduced the rheobase (Figure 4A, CON: 0.24 ± 0.02 nA, n =28; NMD: 0.17 ± 0.02 nA, n =41; **p < 0.01, Mann–Whitney test), and increased the number of APs in response to 2 times rheobase current stimulation (Figure 4C and D, CON: 1.05 ± 0.34, n =25 neurons; NMD: 2.64 ± 0.49, n = 40 neurons; *p < 0.05, Mann–Whitney test) and 3 times rheobase current stimulation (Figure 4C and D, CON: 1.96 ± 0.11, n =21 neurons; NMD: 3.50 ± 0.50, n = 36 neurons; *p < 0.05, Mann–Whitney test). However, NMD did not significantly alter the AP threshold (Figure 4B, CON: -16.78 ± 2.02 mV, n = 27 neurons; NMD: -18.37 ± 1.13 mV, n = 41 neurons). These data further support previous conclusion that excitability of colon specific DRG neurons was enhanced after NMD treatment. We next defined the role for NaHS on TL DRG neurons from healthy control rats. Incubation of these neurons with freshly prepared NaHS (250 μM) for 3 min significantly decreased the rheobase (Figure 4A, PRE: 0.18 ± 0.03 nA; NaHS: 0.08 ± 0.01 nA; n = 13 cells; **p < 0.01, paired sample sign test) and hyperpolarized the threshold of action potentials (Figure 4B, PRE: -22.11 ± 3.63 mV; NaHS: -33.53 ± 2.34 mV; n = 13 cells; *p < 0.05, paired sample *t*-Test). Additionally, application of NaHS also increased the numbers of APs evoked by 2 times and 3 times rheobase current stimulation in TL DRG neurons from healthy control rats. The average numbers of APs after NaHS application were significantly higher than those before NaHS application (Figure 4D, Pre: 1.54 ± 0.22; NaHS: 2.85 ± 0.44; n = 13 cells, 2X rheobase; **p < 0.01, paired sample sign test. Pre: 1.11 ± 0.11; NaHS: 3.33 ± 0.82; n = 9 cells, 3X rheobase; *p < 0.05, paired sample sign test). These data suggest that H_2_S donor NaHS mimics the effect of NMD.

### Administration of NaHS reduces distention threshold

**Figure 5:**
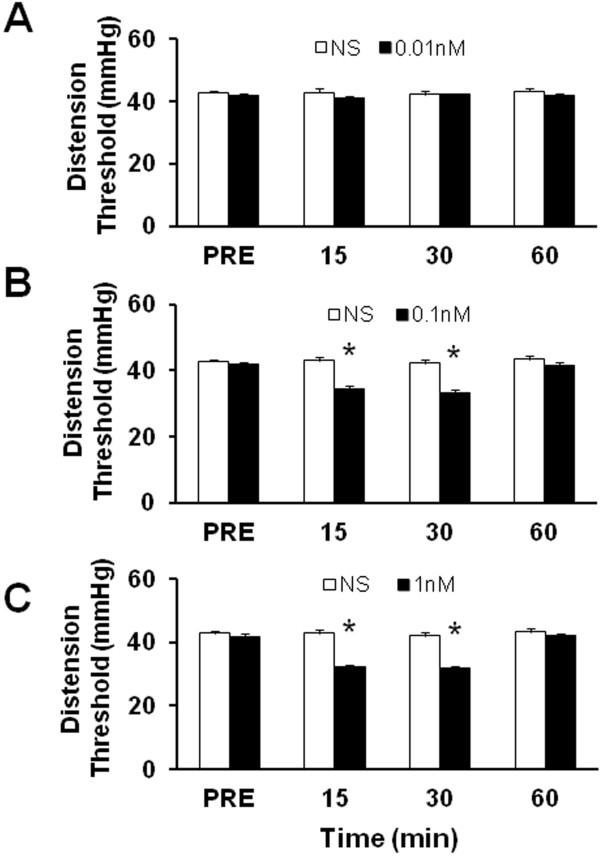
**NaHS reduced distention threshold.** Intrathecal (i.t.) injection of NaHS significantly attenuated the distention threshold in a dose-dependent fashion. NaHS with different concentrations was injected in a volume of 30 μl. The same volume of normal saline (NS) was injected (i.t.) as control. (**A**) NaHS at 0.01 nM did not produce any effect on distention threshold compared with NS group. (**B**) NaHS at 0.1 nM significantly reduced distention threshold compared with NS group. (**C**) NaHS at 1 nM remarkably reduced distention threshold compared with NS group. n=6 rats for each group. *p<0.05.

If H_2_S generated endogenously contributes to the initiation and maintenance of visceral hyperalgesia in NMD animals, then application of exogenous H_2_S to healthy rats should mimic the effects of NMD. Therefore, we applied NaHS to healthy rats and assessed behavioral responses. Intrathecal administration of NaHS, which is used to mimic the CBS production of H_2_S, led to a significant decrease in distention threshold in normal rats, in a dose-dependent manner (Figure 5). Application of 0.01 nM NaHS in 30 μL did not produce any effect on distention threshold (Figure 5A). Application of 0.1 nM (Figure 5B) and 1 nM (Figure 5C) NaHS in 30 μL significantly reduced distention threshold (n=6 for each group, * p < 0.05, Tukey post hoc test following two-way repeated measures ANOVA), and the effect of drug application lasted for at least 30 min. The maximal inhibitory rate was 21% and 25% for 0.1 nM and 1 nM NaHS, respectively. These data demonstrate that H_2_S produces acute hyperalgesic effect in healthy rats, which partially mimics the effect induced by NMD.

### NMD promotes nuclear translocation of NF-κB

**Figure 6:**
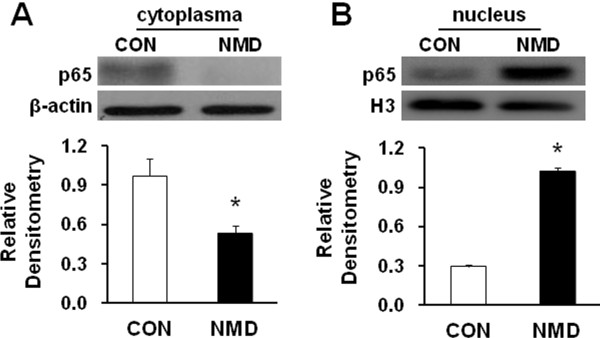
**Effect of NMD on p65 translocation.** Colon-innervating DRGs (T_13_-L_2_) were harvested from control (CON) and NMD rats. After cell fraction, 20 μg of nuclear and 40 μg of cytosolic fractions were used for p65 western blotting. (**A**) Original Western blots demonstrating expression of p65 subunit of NF-κB protein (upper) in cytosolic fractions from control (left) and NMD (right) rats. β-Actin (lower panel) was used as a loading control. Quantification of the bands by densitometry showing that the content of p65 in the cytoplasm was significantly lower in NMD group than in CON group. n=5 rats for CON, n=4 rats for NMD; *P<0.05. (**B**) Original Western blots demonstrating expression of p65 subunit (upper) in nuclear fractions from control (left) and NMD (right) rats. Histone 3 (H3, lower panel) was used as a loading control. Quantification of the bands by densitometry showing that the content of p65 in the nuclear was significantly higher in NMD group than in CON group. n=3 samples for each group, 4 rats in each sample; *P<0.05. Thus, NMD promotes p65 translocation from cytoplasm to nucleus in colon-innervating DRGs.

Because NF-κB plays an important role in inflammatory and neuropathic pain [15,16], we next determined whether NF-kB plays a role in NMD-induced visceral hyperalgesia. Since p65 is a major component in the NF-kB complex activation and predominantly expressed in small- and median-sized DRG neurons [34], we selected to examine p65 translocation to the nucleus by Western blotting. We first examined the distribution of p65 in cytoplasm and nuclear in DRGs from rats 7 weeks after NMD and from age-matched control rats. In NMD rats, the p65 in cytoplasm is greatly decreased compared with the controls (Figure 6A, * p < 0.05, two sample t test). The relative densitometry of p65 expression in cytoplasm was 0.97±0.13 (n=5) and 0.53±0.06 (n=4) for CON and NMD, respectively. NMD reduced cytosolic p65 expression by 45.32%. However, the relative densitometry of nuclear fraction of p65 was more in NMD rats than in controls (Figure 6B, * p <0.05, two sample t test). The relative densitometry of p65 expression in the nuclear was 0.29±0.02 and 1.02±0.03 for CON and NMD (n=3 samples for each group,4 rats in each sample), respectively. NMD increased nuclear p65 expression by 247.98%. Thus, translocation of p65 from the cytosol into the nucleus was evident after NMD.

### p65 Inhibitor PDTC reverses upregulation of CBS expression and mitigates visceral hyperalgesia

**Figure 7:**
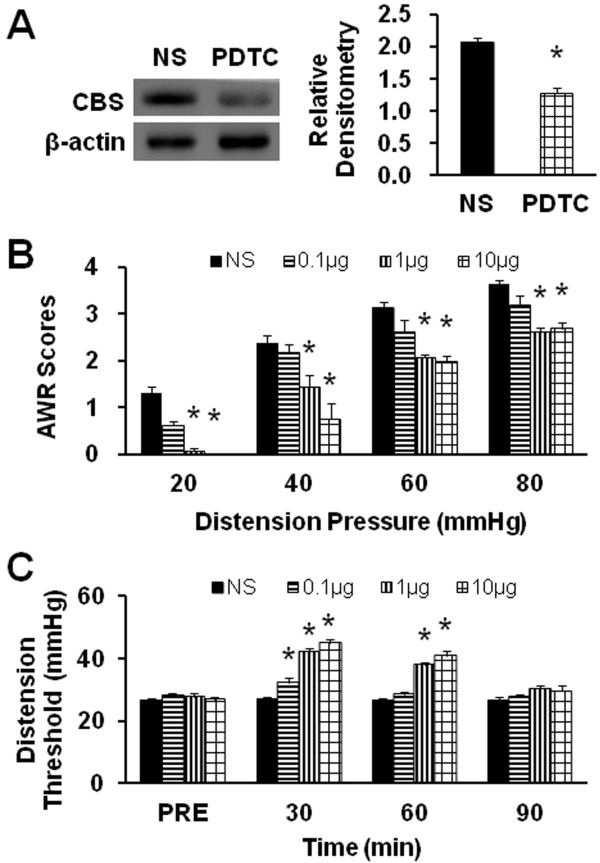
**PDTC reduced CBS expression and attenuated visceral hypersensitivity.** (**A**) Original Western blots demonstrating expression of CBS protein (upper) from NS (left) and PDTC (right) treated rats. β-Actin (lower panel) was used as a loading control. Quantification of the bands by densitometry showing that the intrathecal injection of PDTC at 1 μg once a day for consecutive 7 days remarkably inhibited CBS expression. n=3 rats for each group. *P<0.05. (**B**) Intrathecal injection of different doses of PDTC reduced the AWR scores compared with NS treatment, in a dose-dependent manner. n=8 rats for each group. *P<0.05. (**C**) Time course of PDTC effect. Administration of PDTC greatly enhanced the distension threshold on NMD rats for ~60 min compared with NS-treated rats. n=8 rats for each group. *P<0.05.

Since activation of NF-κB was observed, the possibility of kappaB-dependent gene expression in response to exposure to NMD was investigated. To determine the relationship between NF-κB and CBS upregulation, PDTC, an inhibitor of p65, was used in this study. PDTC (1 μg in 30 μl) was intrathecally injected daily for consecutive 7 days in NMD rats. NS injection was used as control. Proteins were isolated from T1_3_-L_2_ DRGs of those rats. After PDTC treatment, the level of CBS expression was decreased markedly when compared with NS treatment group (Figure 7A, PDTC/NS =0.62, n = 3 for each group; *p < 0.05, two sample t-test). Thus, inhibition of NF-κB abrogates CBS upregulation by NMD.

We next examined the effect of PDTC treatment on visceromotor responses to CRD. Three different doses were used in this experiment. PDTC attenuated AWR scores in NMD rats in a dose- and time- dependent manner (Figure 7B and C). Two doses (1μg and 10 μg in 30 μl) attenuated AWR scores at different distention pressures when compared with NS group (Figure 7B, n=8 for each groups, * p<0.05, Tukey post hoc test following Kruskal-Wallis ANOVA). The maximal effect was at 10 μg/rat. The time course of PDTC effect was also examined. As shown in Figure 7C, the distention threshold was measured after NS or PDTC treatment. Three doses of PDTC significantly reduced distention threshold 30 min after PDTC injection while only two doses (1 and 10 μg) remarkably reduced the distention threshold 60 min after PDTC injection when compared with NS injection (Figure 7C, n=8 for each groups, * p<0.05, Tukey post hoc test following two-way repeated measures ANOVA). PDTC effect disappeared 90 min after treatment.

## Discussion

Our present study showed that NMD produced visceral hypersensitivity in adult Sprague–Dawley rats, which was manifested as a reduction in distention threshold in response to CRD in adult rats compared with age-matched controls. The reduced distention threshold lasted for ~6 weeks (Figure 1). As an extension from a previous study, we showed that NMD reduced the distention threshold to elicit visceromoter responses to CRD [35]. Our result is consistent with previous studies and supported the idea that early life stress could trigger visceral hyperalgesia and colonic dysfunction [2,36-38].

To elucidate the mechanism by which NMD enhanced visceral sensitivity, we analyzed expression of the endogenous hydrogen sulfide producing enzyme CBS in colon-innervating DRGs. We found that NMD greatly upregulated CBS enzyme expression (Figure 1). This upregulation was well paralleled with reduced distention threshold in terms of time course, indicating a close correlation between CBS expression and visceral hypersensitivity in this model. To further confirm the involvement of CBS in NMD-induced visceral pain, AOAA, an inhibitor of CBS, was administrated. AOAA remarkably attenuated the AWR scores in NMD rats in a time- and dose-dependent manner. This is consistent with our previous report that hydroxylamine (HA), another CBS inhibitor, significantly attenuated the AWR scores in rats with neonatal colonic inflammation [8]. The reason for using AOAA but not HA in the present study is because HA has a COX-1 inhibitory action [39]. Of note is that AOAA did not produce any effect in age-matched healthy control rats, suggesting that AOAA had a specific effect under visceral pain conditions. AOAA treatment remarkably reduced excitability of colon-innervating DRG neurons (Figure 3), as was evidenced by our findings that AOAA significantly increased the rheobase and decreased number of APs evoked by 3 times current stimulation. In this study, AOAA treatment did not produced any effect on resting membrane potentials of colon specific DRG neurons. Thus, a increase in the rheobase and decrease in the number of action potentials evoked by current stimulation is not due to alteration of resting membrane potentials. This raised a possibility that functions of voltage-gated Na^+^ and/or K^+^ channels may be modified following AOAA treatment because these channels are major determinant factors for rheobase and action potential firing. Although the detailed mechanisms have yet to be elucidated in future, our findings indicate that the reduction in visceromotor responses to CRD by AOAA application may be attributed to the reduced excitability of colon-innervating DRG neurons.

To further confirm the role for CBS-H_2_S signaling, we examined the effect of H_2_S on neuronal excitability of colon-innervating DRG neurons. NaHS, a donor of H_2_S, was added to mimic CBS production of H_2_S [40]. NaHS greatly enhanced excitability of colon-innervating DRG neurons acutely isolated from healthy control rats (Figure 4). This was evidenced by our findings that NaHS significantly decreased the rheobase, hyperpolarized the AP threshold and increased the numbers of APs evoked by 2 times and 3 times current stimulation. Interestingly, in this study we also found that NMD markedly reduced the rheobase and increased the number of action potentials evoked by 2 times and 3 times rheobase current stimulation of colon-innervating DRG neurons, a phenomena which is very similar to NaHS effects. Furthermore, intrathecal injection of NaHS significantly decreased the distention threshold in response to CRD in healthy control rats (Figure 5). Together, these results suggest that CBS-H_2_S signaling was involved in the development of visceral pain induced by NMD.

It was noteworthy that CBS enzyme upregulation was correlated with the increase in the amount of NF-κB (p65) in the cell nucleus. NF-κB, a pivotal player in inflammatory responses and neuropathy, is constitutively expressed in the primary sensory neurons and glial cells in dorsal root ganglion [41]. It is known that NF-κB participates in pain processes, an effect attributed to the effect of NF-κB on gene transcription. NF-κB is bound to the inhibitory protein IκB, which retains NF-κB within the cytosol thus preventing its transcriptional activity. In this study, we demonstrated that the amount of p65 protein in the cell cytoplasma was reduced by ~45% while the amount of p65 protein in the cell nucleus was enhanced by ~248% after NMD (Figure 6). This suggests that NMD promoted translocation of p65 from cytoplasma to nucleus. This NF-κB activation is critical in the induction of CBS expression since PDTC, an inhibitor for p65, significantly reduced CBS expression (Figure 7A). More importantly, NF-κB translocation is involved in the induction of visceral hypersensitivity in NMD rats since application of PDTC markedly attenuated AWR scores in a dose- and time-dependent fashion (Figure 7B and C). Collectively, our findings suggest that PDTC may exert antinociceptive effect by inhibiting NF-kB-mediated CBS expression. It is of note that NMD-evoked dramatic increase in p65 protein expression in nucleus may not be explained simply by the enhanced translocation. An upregulation of total p65 protein expression cannot be ignored since the 45% reduction in cytoplasma may not account for the 248% increase in nucleus. Regulatory mechanism at transcriptional level should be considered. Future studies are needed to determine whether NF-KB mediated-CBS upregulation is a direct transcriptional regulation or an indirection interaction. Nevertheless, enhanced translocation might play a critical role in mediating CBS expression. Although no common second messenger has been identified, most NF-κB activating signals can be inhibited by antioxidants because critical steps such as protein phosphorylation and binding of transcription factors to consensus sites on DNA are regulated by the intracellular redox status. Future researches are needed to investigate this mechanism since H_2_S production was increased in DRG cells due to its ability to activate NF-κB by increasing the intracellular redox state.

Taken together, p65 is a signal transducer that modulates CBS-H_2_S signaling pathway, which in turn affects colon-innervating sensory neuron excitability and thereby contributing to the chronic visceral pain seen in NMD rats. Thus, NF-κB inhibitors may be promising candidates for treating visceral pain in patients with functional gastrointestinal disorders such as IBS.

## Abbreviations

AOAA: O-(Carboxymethyl) hydroxylamine hemihydrochloride; AWR: Abdoninal withdrawal reflex; CRD: Colorectal distention; CVH: Chronic visceral hyperalgesia; DT: Distention threshold; H_2_S: Hydrogen sulfide; IBS: Irritable bowel syndrome; NF-kB: Nuclear factor kappa B; NMD: Neonatal maternal deprivation; PDTC: Pyrrolidine dithiocarbamate.

## Competing interests

The authors declare that they have no competing interests.

## Authors’ contributions

Lin Li: Performed experiments, analyzed data, prepared figures and the manuscript. Ruihua Xie: Performed experiments, analyzed data, prepared figures and the manuscript. Shufen Hu: Performed experiments and prepared figures Yongmeng Wang: Performed experiments and prepared figures. Tianzhu Yu: Performed experiments Ying Xiao: Analyzed data, prepared figures Xinghong Jiang: Interpret data and prepared the manuscript. Jianguo Gu: Interpret data and edited the manuscript. Chuang-Ying Hu: Designed experiments and prepared the manuscript Guang-Yin Xu: Designed experiments and edited the manuscript.
